# Deworming women of reproductive age during adolescence and pregnancy: what is the impact on morbidity from soil-transmitted helminths infection?

**DOI:** 10.1186/s13071-021-04620-w

**Published:** 2021-04-23

**Authors:** Carolin Vegvari, Federica Giardina, Sumali Bajaj, Veronica Malizia, Robert J. Hardwick, James E. Truscott, Antonio Montresor, Sake J. de Vlas, Luc E. Coffeng, Roy M. Anderson

**Affiliations:** 1 Department of Infectious Disease Epidemiology, London Centre for Neglected Tropical Disease Research (LCNTDR), Imperial College London, St Mary’s Campus, Praed Street, London, W2 1PG UK; 2 Department of Infectious Disease Epidemiology, School of Public Health, Faculty of Medicine, Imperial College London, St Mary’s Campus, Praed Street, London, W2 1PG UK; 3 Medical Research Council Centre for Global Infectious Disease Analysis, Department of Infectious Disease Epidemiology, School of Public Health, Imperial College London, London, UK; 4 Department of Public Health, Erasmus MC, University Medical Center Rotterdam, Rotterdam, The Netherlands; 5 The DeWorm3 Project, The Natural History Museum of London, London, SW7 5BD UK; 6 Department of Control of Neglected Tropical Diseases, World Health Organization, Geneva, Switzerland

## Abstract

**Background:**

Soil-transmitted helminths (STHs) are a major cause of poor health in low- and middle-income countries. In particular, hookworm is known to cause anaemia in children and women of reproductive age (WRA). One goal of the World Health Organization’s (WHO) 2030 roadmap for neglected tropical diseases is to reduce STH-related morbidity in WRA. As a minimal intervention, the WHO recommends deworming adolescent girls annually during human papilloma virus vaccination programmes and WRA during pregnancy and lactation. These routine interventions are low cost and can be implemented even by the most basic health services in endemic countries. In this study we use a cohort model to investigate the potential impact on STH-related morbidity in WRA.

**Results:**

Annual deworming treatment of adolescent girls reduces the prevalence of moderate- and heavy-intensity infections in this age group by up to 60% in moderate transmission settings and by 12–27% in high transmission settings. Treatment of WRA during pregnancy and lactation on its own has a small (< 20%) but significant effect on morbidity although it does not lead to the achievement of the morbidity target (< 2% moderate- to high-intensity infections) in this age group. However, depending on the age-intensity profile of infection, which may vary geographically, and assumptions on the density-dependence of egg production by fertilised female worms, continued school-based treatment may be able to reduce the force of infection acting on WRA, both through an indirect effect on the overall population-based force of infection and* via* reducing the burden of infection as children age and move into the WRA age classes. As a result, morbidity in WRA may be eliminated.

**Conclusion:**

While deworming during pregnancy and lactation does not lead to the achievement of the morbidity target in WRA and its efficacy may vary by setting, it is still expected to be beneficial for maternity and child health. Monitoring of any WRA-based intervention is recommended to evaluate its effectiveness.
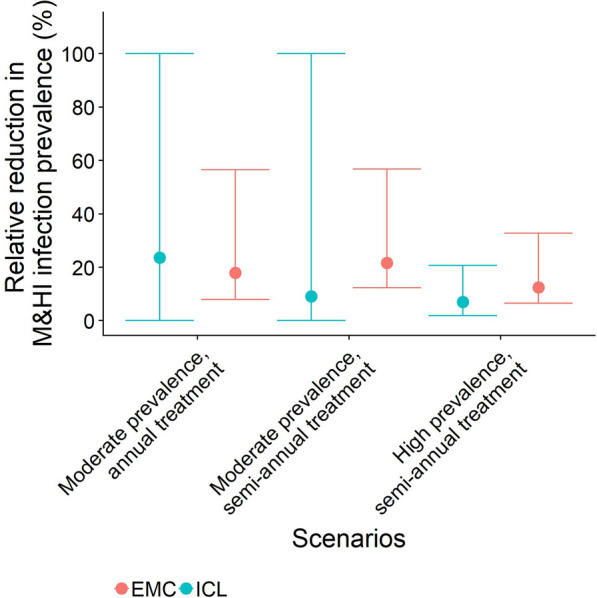

## Background

Soil-transmitted helminths (STH) are tropical parasites that affect 1.7 billion people worldwide [1]. Hookworm (*Ancylostoma duodenale* and *Necator americanus*) in particular can cause significant morbidity, mainly anaemia. The severity of anaemia has been observed to increase in individuals with moderate- and high-intensity (M&HI) infection [2, 3]. In hookworm, M&HI infection is defined as > 2000 eggs per gram (epg) faeces measured by a Kato-Katz faecal smear test [4]. Anaemia can lead to stunted development during childhood, has a negative impact on the health of women of reproductive age (WRA, 18–49 years) and can contribute to adverse pregnancy outcomes [3]. Current treatment guidelines recommend school-based (SB) deworming of pre-school age children (pSAC, 2–4 years) and school-age children (SAC, 5–14 years) through mass drug administration (MDA), which is given annually in moderate-prevalence settings (20–50%) and semi-annually in high-prevalence settings (> 50%). In addition, as part of the new 2030 roadmap to achieve morbidity control of STH, the World Health Organization (WHO) recommends the treatment of WRA and adolescent girls (15–19 years) with one dose of 400 mg albendazole [5]. Benzimidazole drugs for the treatment of STHs are currently only donated for pSAC and SAC, but not for adults. In addition, costs of a community-wide distribution can be significant. Therefore, community-wide treatment of WRA is not economically feasible for most low- and middle-income countries (LMICs) with the presently available resources.

To minimise the most adverse impact of anaemia on WRA during pregnancy and lactation, the WHO proposes an economically feasible strategy to treat WRA when they come into contact with the health system at prenatal and postnatal clinics. In addition, the WHO recommends preventive chemotherapy treatment of adolescent girls during annual human papilloma virus (HPV) vaccine programmes. The rationale behind this strategy is that after years of SB treatment, the prevalence of M&HI infections in children/adolescents is low, reported to be on average 2% after 5 years of MDA in a review of studies from 17 countries [6]. Consequently, young women in most countries, where annual SB deworming is implemented at effective coverage (75% for STHs), are expected to start their reproductive lifespan with no or low worm burden so that young girls leaving school do not immediately require MDA. The hypothesis is that infection intensity needs time to build up and that treating adolescent girls and WRA (new strategy), who have passed through SB-deworming as children, at regular intervals during pregnancy and lactation could prevent them from developing M&HI infections. We use a simple cohort model and the force of infection (FoI) derived from two independently developed individual-based stochastic models of STH infection to investigate this hypothesis. The FoI is the per capita rate at which susceptible individuals acquire worms [7]. We choose a cohort model because it makes it easier to follow a cohort of women under controlled conditions compared to the fully stochastic individual-based models which include additional complexities. A cohort model is computationally simpler and fulfils the purpose of our analysis equally well. We focus on hookworm because hookworm-induced anaemia is a recognised cause of pregnancy morbidity and mortality in LMICs and the relationship of infection intensity and morbidity is better understood and documented for this than for other STHs [8].

## Methods

### Cohort model

We use a cohort model that compares the currently implemented strategy (SB treatment of pSAC and SAC) and the new strategy that extends deworming to adolescent girls and WRA during pregnancy and lactation. We simulate a birth cohort of girls/women and express the effectiveness of the new strategy as the reduction of the risk to develop morbidity in adolescent girls and WRA. In the cohort model all girls/women are simulated from age 0 years and are followed up to age 70 years. Other individuals are not simulated in the cohort model. We apply external FoIs to the cohort of girls/women, the reason being that the FoI cannot be inferred from the cohort model itself because the reservoir of infection for STH transmission is contributed to by the entire community, and not just by the cohort of interest. The FoIs have been extracted from two different individual-based stochastic transmission dynamics models of STH infection developed at Imperial College London (ICL) [9, 10] and Erasmus MC (EMC), Rotterdam [11]. The cohort model itself does not take into account reductions in the FoI due to treating WRA. This assumption is based on the consideration that not all women are pregnant at the same time, and even if 80% of pregnant women visit maternity health centres and receive deworming treatment, this corresponds to very low annual adult coverage that would not contribute much to depleting the infectious reservoir.

### Cohort model assumptions

The cohort model is based on the following assumptions:

- Women with M&HI infections suffer morbidity from hookworm infection.
- SB treatment only happens when women are aged 2–14 years.
- Coverage of 75% for pSAC and SAC. Non-access to treatment is assumed to be random at an individual level, i.e. independent of an individual’s infection status and history of participating in deworming.
- Deworming of adolescent girls (15–19 years) happening through annual SB programmes and during HPV vaccination programmes. It is assumed that annual HPV programmes will target the whole cohort of girls aged 15–19, and although each adolescent girl is only vaccinated once, all girls of the cohort will attend the programme. This provides an opportunity to treat adolescent girls for STH on an annual basis. The assumed treatment coverage in this cohort is 75%. Non-access to treatment is assumed to be random.
- Deworming during pregnancies occurs twice, during the second and third trimester, at prenatal clinics. Furthermore, we assume on average four pregnancies per woman over the reproductive lifespan (15–50 years of age) [12]. Deworming happens one more time during the year after pregnancy at maternity health clinics. Further, 80% of pregnant and lactating women make use of prenatal and postnatal clinics. This figure is based on estimated coverage of pregnant women through existing health services [13]. Access to maternity services and to treatment are assumed to be random at the level of the individual.

### Stochastic, individual-based model

Details of the two transmission dynamics models have been published previously [9, 11, 14]. Unlike the cohort model, the fully stochastic individual-based STH transmission models represent age-structured human populations. The age structure in both models follows demographic data from Kenya taken from the 2003 Demographic and Health Surveys [15]. Each simulated year, pSAC and SAC receive SB deworming treatment. Each transmission dynamics model was run 100 times, the FoIs were estimated in monthly timesteps, and the mean FoIs of 100 runs over time were applied to the cohort model.

There are two important differences between the ICL and the EMC model. First, the ICL model assumes that the relative exposure to hookworm infections and contribution to the environmental reservoir of infection are the same for all age groups and only depend on the burden of adult worms in an age group [16]. In other words, the FoI is fixed and independent of age. In contrast, the EMC model assumes that the relative exposure and contribution to the environmental reservoir increase linearly from age 0 years until age 10 years and stay constant thereafter [8]. Secondly, both models assume density-dependent saturation of egg production by female hookworm inside the human host. The ICL model assumes that this saturation follows an exponential function [9, 17], whereas the EMC model assumes a hyperbolic saturation function [11]. The distinction between these two is that individuals with large worm burdens in the ICL model contribute much smaller egg/larvae counts into the reservoir of infection than those in the EMC model. Due to the sparse and high-variance data to infer the tails of these egg production functions, the difference between the ICL and EMC models remains an unavoidable modelling uncertainty that necessitates a comparison of the conclusions drawn with both for greater scientific rigor.

### Scenarios simulated using the cohort model

We apply FoIs extracted from the two fully stochastic individual-based STH transmission models where ongoing annual or semi-annual SB treatment has been implemented over the whole time-course of the simulation, 70 years, to obtain a FoI value under treatment for each year of the girls’/women’s lifespan. However, the girls in the cohort only receive SB treatment when they are pSAC or SAC themselves.

We focus on two SB treatment strategies: annual and semi-annual MDA targeting pSAC and SAC for hookworm species. Annual SB treatment is recommended in settings where the prevalence of infection with any STH species is 20–50% (moderate). Semi-annual SB treatment is implemented in settings where the prevalence of infection with any STH species is > 50% (high). We simulate semi-annual treatment for settings with high hookworm prevalence. For settings with moderate prevalence, we simulate both annual and semi-annual treatment, the reasoning being that although the prevalence of hookworm alone may be moderate, the prevalence of all infections, including other STH species, may be high.

For each strategy, we then compare SB treatment only and SB treatment plus treatment of WRA according to the new suggested guidelines (annual treatment of adolescent girls, treatment twice during pregnancy and once during lactation, irrespective of infection prevalence). Each simulated scenario of the cohort model is run for 100 iterations, and the mean prevalence of M&HI infections over time and 95% credible intervals are calculated from the results. Each iteration is run with a seed in a reproducible way so that each iteration of the SB treatment-only scenario pairs with an iteration of the SB plus WRA treatment scenario.

The average prevalence of M&HI infections over all simulated time points for adolescent girls and WRA is calculated for each scenario. Table 1 lists all scenarios considered to be part of this study. The code for the cohort model can be found in the ‘github’ repository (https://github.com/caro-veg/STH_WRA_Morbidity). The parameters that are used in the stochastic individual-based simulations to generate the FoIs are included in Additional file 1: Table S1.

**Table 1:** Overview of simulated treatment scenarios

Baseline prevalence of any hookworm infection	SB treatment only	SB treatment and treatment of WRA
Moderate	Annual SB treatment Semi-annual SB treatment	Annual SB treatment Semi-annual SB treatment
High	Semi-annual SB treatment	Semi-annual SB treatment

SB, School-based; WRA, women of reproductive age

For each baseline prevalence and treatment frequency we compare the currently implemented treatment strategy (SB treatment only) with the new recommended treatment strategy (SB treatment + treatment of WRA). All analyses were done separately with the forces of infection (FoIs) extracted from the Erasmus Medical Centre (EMC) and Imperial College London (ICL) models

The relative reduction in the prevalence of M&HI infections is calculated as follows:

$$\frac{{\text{Prevalence MHI infections with old guidelines}}-{\text{Prevalence MHI infections with new guidelines}}}{{\text{Prevalence MHI infections with old guidelines}}}$$

### Statistical comparison of SB treatment only* vs* SB treatment plus WRA treatment

The average point prevalences for adolescent girls, WRA and adolescent girls combined with WRA are compared between paired simulations of SB treatment only and simulations of SB treatmet plus WRA treatment using the Wilcoxon signed-rank test. The alternative hypothesis is that the prevalence of M&HI infections in adolescent girls and WRA is less in the scenario with the new treatment guidelines.

## Results

### Moderate prevalence settings

**Fig. 1:**
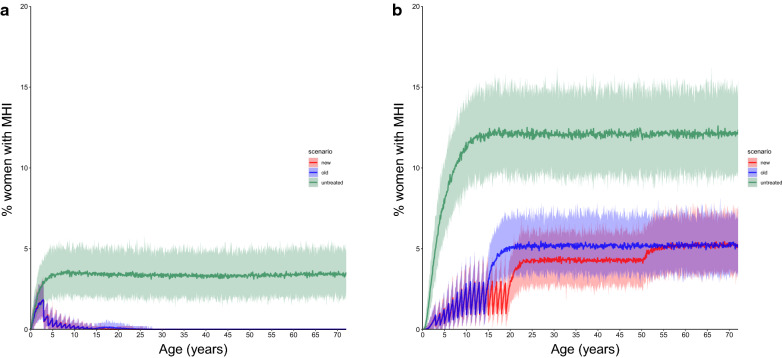
Percentage of girls/women with moderate- and high-intensity infections (*MHI*) infections with aging, with moderate baseline prevalence (20–50%) and annual school-based (SB) treatment. The force of infection (FoI) inputted into the cohort model was extracted from the the Erasmus Medical Centre (EMC) and Imperial College London (ICL) fully stochastic individual-based models with ongoing SB treatment, i.e. FoI decreased over time. Differences between the prevalence of MHI infections between the two models are due to different assumptions on the density-dependent egg production functions and age-dependent exposure to infection. **a** Results with the ICL model, **b** results with the EMC model. Solid lines are the mean, shaded areas are the 95% credible interval. Green indicates no treatment (untreated), blue indicates treatment of pre-school-age children (pSAC) and school-age children (SAC) only (old guidelines), red indicates treatment of pSAC and SAC plus annual treatment of adolescent girls (15–19 years) plus treatment of women during pregnancy and lactation (new suggested guidelines)

**Fig. 2:**
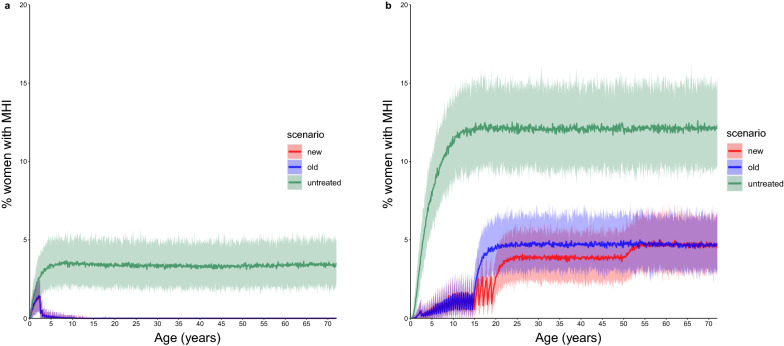
Percentage of girls/women with moderate- and high-intensity infections (*MHI*) with aging, with moderate baseline prevalence (20–50%) and semi-annual SB treatment. The FoI inputted into the cohort model was extracted from the ICL and EMC fully stochastic individual-based models with ongoing SB treatment, i.e. FoI decreased over time. Differences between the prevalence of MHI infections between the two models are due to different assumptions on the density-dependent egg production functions and age-dependent exposure to infection. **a** Results with the ICL model, **b** results with the EMC model. Solid lines are the mean, shaded areas are the 95% credible interval. Green indicates no treatment (untreated), blue indicates treatment of pSAC and SAC only (old guidelines), red indicates treatment of pSAC and SAC plus annual treatment of adolescent girls (15–19 years) plus treatment of women during pregnancy and lactation (new suggested guidelines)

The ICL model predicts that with ongoing annual SB treatment the prevalence of M&HI infections is eliminated in the women followed in the cohort model by age 25 years, if WRA themselves are not treated, and by age 20 years, if treatment of WRA is implemented (Fig. 1; Table 2). The ICL model predicts a reduction of 77.8% (95% credible interval [CI] 59.2–91.9%) in the average point prevalence of M&HI infections in women aged 15–50 years where annual SB treatment is ongoing (Fig. 1; Table 2). For semi-annual treatment, the decline in M&HI infections is even faster: the estimated reduction in the point prevalence of M&HI infections in women aged 15–50 years where semi-annual treatment is implemented is 41% (95% CI 0–100%) (Fig. 2; Table 3). Note that there is large uncertainty around this estimate because the prevalence of M&HI infections in WRA in the ICL model is very low.

**Table 2:** Average prevalences of moderate- and high-intensity infections in women of reproductive age

Model^a^	Age period (years)	SB only, mean (95% credible interval)	SB + WRA, mean (95% credible interval)	% Reduction, mean (95% credible interval)^b^	*P* value
ICL	15–50	0.0216 (0.0142–0.0328)	4.81 × 10^−3^ (2.35 × 10^−3^–8.57 × 10^−3^)	77.8 (59.2–91.9)	< 2.2 × 10^−16^
ICL	15–19	0.101 (0.06–0.141)	0.0173 (3.33 × 10^-3^–0.03)	83.5 (66.7–95.9)	< 2.2 × 10^−16^
ICL	20–50	8.828 × 10^−3^ (3.88 × 10^−3^–0.0127)	2.73 × 10^−3^ (5.54 × 10^−4^–5.57 × 10^−3^)	61.5 (− 22.6 to 100)	< 2.2 × 10^−16^
EMC	15–50	5.06 (4.54–5.46)	3.90 (3.47–4.26)	23.0 (20.1–26.0)	< 2.2 × 10^−16^
EMC	15–19s	4.42 (3.93–4.85)	2.00 (1.70–2.35)	55.2 (50.6–60.3)	< 2.2 × 10^−16^
EMC	20–50	5.16 (4.66–5.59)	4.21 (3.78–4.59)	18.3 (15.0–21.7)	< 2.2 × 10^−16^

Values in table are presented as the mean prevalence (%)

^a^Moderate baseline prevalence (20–50%) and annual SB treatment. The FoI inputted into the cohort model was extracted from the ICL and EMC fully stochastic individual-based models with annual ongoing SB treatment, i.e. FoI decreased over time. In the ICL model prevalences of moderate- and high-intensity infections (M&HI) infections in WRA are well below 1%. Therefore, the reduction in the prevalence of M&HI infections in absolute numbers is negligible and the uncertainty large

^b^The relative reduction is only calculated from time points where the prevalence of M&HI infections in simulations with the old treatment strategy (SB only) is > 0. All time points were used in the statistical comparison.

**Table 3:** Average prevalences of moderate- and high-intensity infections infections in women of reproductive age

Model^a^	Age period (years)	SB only, mean (95% credible interval)	SB + WRA, mean (95% credible interval)	% Reduction, mean (95% credible interval)^b^	*P* value
ICL	15–50	3.42 × 10^−4^ (0–1.43 × 10^−3^)	5.23 × 10^−5^ (0–4.75 × 10^−4^)	41 (0–100)	9.33 × 10^−7^
ICL	15–19	1.97 × 10^−3^ (0–0.01)	3.33 × 10^−4^ (0–3.33 × 10^−3^)	37 (0–100)	1.27 × 10^−6^
ICL	20–50	0 (0–0)	0 (0–0)	0 (0–0)	NA
EMC	15–50	4.56 (4.11–4.97)	3.51 (3.13–3.85)	23.0 (20.5–25.7)	< 2.2 × 10^−16^
EMC	15–19	3.72 (3.29–4.11)	1.75 (1.50–2.01)	53.0 (48.0–58.6)	< 2.2 × 10^−16^
EMC	20–50	4.70 (4.25–5.10)	3.81 (3.41–4.17)	19.0 (16.2–21.9)	< 2.2 × 10^−16^

Values in table are presented as the mean prevalence (%)

^a^Moderate baseline prevalence (20–50%), semi-annual SB treatment. The FoI inputted into the cohort model was extracted from ICL and EMC fully stochastic individual-based models with ongoing semi-annual SB treatment, i.e. FoI decreased over time. In the ICL model prevalences of M&HI infections in WRA are below 1%. Therefore, the reduction in the prevalence of M&HI infections in absolute numbers is negligible and the uncertainty large

^b^The relative reduction is only calculated from time points where the prevalence of M&HI infections in simulations with the old treatment strategy (SB only) is > 0. All time points were used in the statistical comparison

In contrast, in the EMC model the reduction in the FoI from SB treatment is much less pronounced. A reduction of the FoI occurs over the first 10 years after which a new equilibrium FoI under treatment is reached. The reason for this is that in the EMC model, pre-control infection levels in children are lower than those of adults (i.e. they are considered to contribute and be exposed to transmission differentially). The EMC model predicts a reduction of 23.0% (95% CI 20.1–26.0%) in the average point prevalence of women aged 15–50 years where annual SB treatment is ongoing (Fig. 1; Table 2) and a reduction of 23.0% (95% CI 20.5–25.7%) where semi-annual SB is ongoing (Fig. 2; Table 3). The predicted relative reduction in the average point prevalence of adolescent girls aged 15–19 years is greater, 55.2% (95% CI 50.6–60.3%), where annual SB treatment is implemented and 53.0% (95% CI 48.0–58.6%) where semi-annual SB treatment is implemented. For women aged 20–50 years, the EMC model predicts a relative reduction by treatment during pregnancy or lactation in the average point prevalence of M&HI infections of 18.3% (95% CI 15.0–21.7%) where annual SB treatment is implemented and of 19.0% (95% CI 16.2–21.9%) where semi-annual SB treatment is implemented (Figs. 1, 2; Tables 2, 3).

### High-prevalence settings

**Fig. 3:**
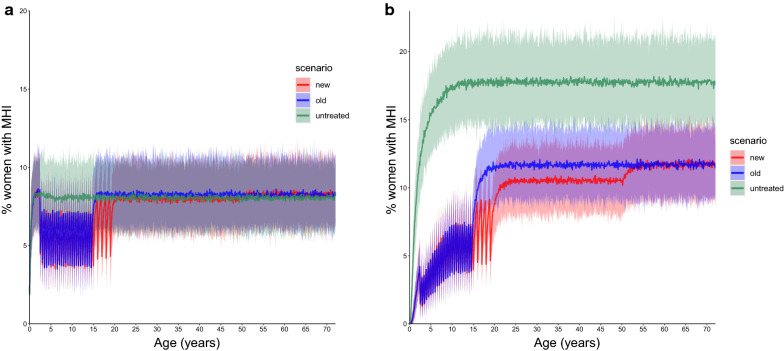
Percentage of girls/women with moderate- and high-intensity infections (*MHI*) with aging, with high baseline prevalence (> 50%) and semi-annual SB treatment. The FoI inputted into the cohort model was extracted from the ICL and EMC fully stochastic individual-based models with ongoing SB treatment, i.e. FoI decreased over time. Differences between the prevalence of MHI infections between the two models are due to different assumptions on the density-dependent egg production functions and age-dependent exposure to infection. **a** Results from ICL model, **b** results from EMC model. Solid lines are the mean, shaded areas are the 95% credible interval. Green indicates no treatment (untreated), blue indicates treatment of pSAC and SAC only (old guidelines), red indicates treatment of pSAC and SAC plus annual treatment of adolescent girls (15–19 years) plus treatment of women during pregnancy and lactation (new suggested guidelines)

The ICL model predicts a relative reduction in the average prevalence of M&HI infection in WRA aged 15–50 years of 5.11% (95% CI 2.03–7.62%) with ongoing semi-annual SB treatment (Fig. 3; Table 4). For adolescent girls (15–19 years), this reduction was 16.8% (95% CI 10.7–23.6%), while for women aged 20–50 years it was 3.17% (95% CI 0.30–5.70%).

**Table 4:** Average prevalences of moderate- and high-intensity infections infections in women of reproductive age

Model^a^	Age period (years)	SB only, mean (95% credible interval)	SB + WRA, mean (95% credible interval)	% Reduction, mean (95% credible interval)	*P* value
ICL	15–50	8.24 (7.72–8.79)	7.82 (7.35–8.30)	5.11 (2.03–7.62)	< 2.2 × 10^−16^
ICL	15–19	8.20 (7.56–8.80)	6.82 (6.26–7.35)	16.8 (10.7–23.6)	< 2.2 × 10^−16^
ICL	20–50	8.25 (7.77–8.82)	7.99 (7.50–8.48)	3.17 (0.30–5.70)	< 2.2 × 10^−16^
EMC	15–50	11.5 (10.8–12.2)	10.0 (9.29–10.6)	13.1 (11.6–14.6)	< 2.2 × 10^−16^
EMC	15–19	10.5 (9.77–11.2)	7.29 (6.60–7.88)	30.8 (27.3–34.5)	< 2.2 × 10^−16^
EMC	20–50	11.7 (11.0–12.3)	10.5 (9.77–11.1)	10.4 (8.52–12.2)	< 2.2 × 10^−16^

Values in table are presented as the mean prevalence (%)

^a^High baseline prevalence (> 50%) The FoI inputted into the cohort model was extracted from the ICL and EMC fully stochastic individual-based models with ongoing SB treatment, i.e. FoI decreased over time

**Fig. 4:**
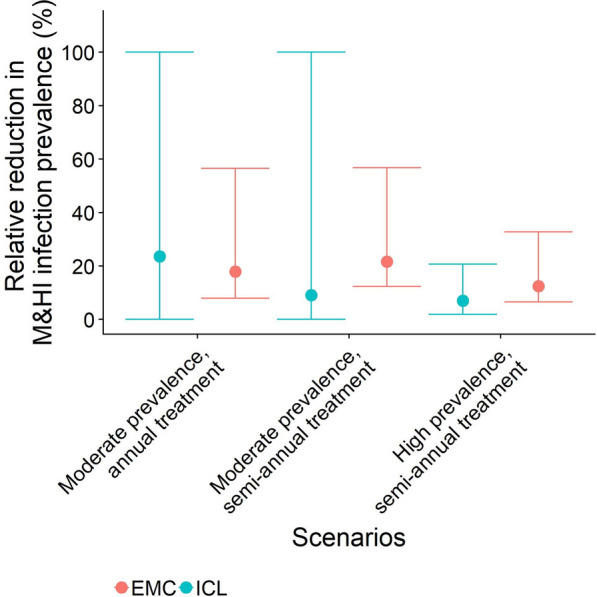
Relative reduction in the prevalence of moderate- and high-intensity infections (*M&HI*) in WRA (15–50 years) with the new treatment strategy (treating school-aged children plus treating adolescent girls annually, treating women twice during pregnancy and once during lactation) compared to the prevalence of M&HI infections in WRA with the old treatment strategy (treating only school-aged children, no treatment of WRA). The FoI inputted into the cohort model was extracted from the ICL and EMC fully stochastic individual-based models with ongoing SB treatment, i.e. FoI decreased over time. Results were obtained from 100 iterations of a cohort model of 500 women run for each scenario. Treatment frequency depended on the transmission setting, according to World Health Organization guidelines. Error bars represent 95% credible intervals

The EMC model predicts a relative reduction in the average point prevalence of WRA (15–50 years) of 13.1% (95% CI 11.6–14.6%) with ongoing SB treatment. The relative reduction in the average prevalence of M&HI infections in adolescent girls and in women aged 20-50 years is 30.8% (95% CI 27.3–34.5%) and 10.4% (95% CI 8.52–12.2%), respectively, according to the EMC model (Fig. 3; Table 4). The results for the cohort model simulations are summarised in Fig. 4.

## Discussion

The results obtained from the cohort model using FoI from two different individual-based stochastic models of STH transmission suggest that annual deworming treatment of adolescent girls reduces the prevalence of M&HI infections in this age group by up to 60% (EMC model) in moderate transmission settings and by 12–27% (both EMC and ICL models) in high transmission settings. The reductions in moderate transmission settings predicted by the ICL model are highly variable. Overall, the reductions are comparable to the impact of SB treatment of pSAC/SAC. The results also suggest that treatment of WRA during pregnancy and lactation reduces the prevalence of M&HI infections by a small but significant fraction of < 20%. In moderate prevalence settings where infection levels are uniformly distributed over age (ICL model), reductions of the FoI over time achieved by ongoing SB treatment alone can prevent morbidity in WRA and, consequently, deworming of WRA is not necessary. However, for highly endemic settings (both models) and settings where adults host the majority of the hookworm population (EMC model), morbidity in WRA will persist at low levels during SB treatment, which allows for (some) additional benefit of targeted deworming of WRA.

The lower impact of treatment during pregnancy and lactation on the prevalence of M&HI infections compared to annual treatment during adolescence or SB treatment is expected, as it corresponds to low-coverage treatment in adult women. Assuming an average of four pregnancies per woman, each woman receives treatment on average every 7–8 years. This frequency is unlikely to completely prevent re-infection and the re-establishment of M&HI infections between treatments.

The differences in the predictions between the two models can be primarily explained by the different age-intensity profiles of infection in the two models and different assumptions on the form of density-dependent egg production by adult female worms. The same factors also explain the differences in baseline prevalences of M&HI infections between the two models (green line in Figs. 1, 2, 3). The ICL model assumes that exposure to infection is constant over all age groups, such that the worm burden rises to a plateau as people age, as observed in the recent Tumikia study data from Kenya [16]. In contrast, the EMC model assumes that exposure and contribution are 0 at age 0 months, increase linearly up to age 10 years and stay constant thereafter. This produces a pattern of infection intensity that increases with age up to the age of about 15 years, matching some previously published age-intensity profiles from various countries [8]. Accordingly, SB treatment has a greater impact on the FoI in the ICL model than in the EMC model in moderate transmission settings. As a result, the ICL model predicts that the prevalence of M&HI infections in WRA is very low and declines over time from SB treatment alone.

A second explanation for the differences between predictions of the two models relates to density dependence in female worm fecundity, which describes how the egg production per female worm declines as the number of worms in a host increases [18]. The EMC model assumes a hyperbolic saturation of the density-dependent fecundity of female worms inside the host, while the ICL model assumes that the density-dependent fecundity of female worms decays in an exponential manner with negative exponent to almost zero as worm burdens rise to high levels [18]. The two functions are similar for low and moderate worm burdens, but different for high worm burdens (eggs produced per female worm decline more rapidly with exponential saturation). As a result, in individuals with a high worm burden, female hookworms produce more eggs in the EMC model than in the ICL model. Consequently, killing the same number of worms by treatment leads to a greater reduction in epg in the EMC model compared to the ICL model. Moreover, because in the ICL model density-dependent processes lead to a reduction in the total number of eggs produced inside a host when worm burdens are very high, a reduction in the worm burden following treatment (and re-infection) can result in higher egg production inside a host. This explains why the impact on the prevalence of M&HI infections in high transmission settings is less in the ICL model than in the EMC model.

In the simulations, we assumed effective treatment coverage of 75% and random access to treatment. Not all countries may achieve this, and non-access to treatment is likely non-random in reality. However, here our focus is on comparing two different treatment strategies. Provided that all assumptions are the same for simulations of the different scenarios that we compare, the results will be informative with respect to the treatment strategies.

When girls/women stop receiving regular SB MDA at effective coverage each year (at age 14 years according to the old guidelines, at age 18 years according to the new guidelines), the prevalence of any infection and specifically the prevalence of M&HI infection are expected to be low in the cohort directly after treatment stops. How long resurgence of prevalences to endemic equilibrium levels will take depends on the worm life expectancy (2–3 years in the case of hookworm) and whether or not treatment has lowered the prevalence of any infection so much that it is close to the transmission breakpoint. The transmission breakpoint is the prevalence below which transmission of the parasite cannot be sustained and the parasite population becomes extinct. If the latter has been achieved, models predict that resurgence of infection can take many years and to depend on the movements of people who can re-introduce infective stages into the environments where transmission interruption has been achieved [10, 19]. If the prevalence of any infection is not close to the transmission breakpoint after treatment, the time to resurgence of infection in the population will be about equivalent to the worm life expectancy, i.e. 2–3 years. In the cohort model, we only look at the prevalence of infection in a cohort of girls/women and not in the background population that they are embedded in. Consequently, we cannot conclude from the results of the cohort model on its own if the transmission breakpoint has been reached or not. In the absence of any treatment (SB treatment or treatment of WRA), the prevalence and the prevalence of M&HI infections in the cohort at any age is always higher than if treatment at effective coverage is implemented (Additional file 2: Figure S1).

If treatment has not eliminated hookworm transmission, there will be a FoI that causes a resurgence of infection between treatments, but not back to the endemic equilibrium levels that would be observed in the absence of treatment (compare spikes between treatments and equilibrium prevalence of M&HI infection in Figs. 1, 2, 3). Resurgence between treatments does not exclude reaching the target of an MDA programme (e.g. < 2% M&HI infections). However, the treatment strategy must be intensive enough for a given intrinsic transmission intensity in a defined setting and must be applied for long enough to reach the target. This has been shown in previous simulation studies [11, 20].

Resurgence of infection may still occur quickly in locations where transmission intensity is high and the infectious reservoir has not been sustainably depleted. For example, Ortu et al. [21] report that a single year of missed treatment led STH resurgence back to baseline levels in an MDA programme for STH in Burundi where the dominant species was *Ascaris lumbricoides*. In a systematic review and meta-analysis of re-infection with STH after MDA, Jia et al. [22] found that re-infection between treatments occurred quickly and that at 12 months after the last treatment, prevalence of *A. lumbricoides* infections had resurged to 94% of baseline levels, that of *Trichuris trichiura* infections to 82% of baseline levels and that of hookworm infections to 56% of baseline levels. Therefore, Jia et al. recommended frequent antihelminthic treatment. Similarly, Gunawardena et al. [23] found a prevalence of M&HI infections of 11.6% 4 years after MDA ceased. Appleton et al. [24] reported rapid resurgence to baseline prevalence levels 4–12 months after treatment in slum-dwelling children in South Africa.

Another study showing that M&HI infections can be acquired rapidly is that of Menzies et al. [25]. The study, set in Ecuador, investigated how quickly children are infected with STH during the first 3 years of life. The authors found that prevalence of any STH infection was about 25% at 3 years of age and that 10–15% of infected 3-year-old children had M&HI infections [25]. This result is in accordance with the prevalence of M&HI infections a few years after treatment in our simulations.

However, rapid resurgence at the local level does not necessarily contradict sustained low prevalences and reaching the WHO 2030 morbidity target at the country level. At the country level, many locations, especially those with low to moderate baseline prevalences, could successfully reach morbidity control, and prevalences could be brought down to levels near the transmission breakpoint from where resurgence, if it happens, takes longer [19]. If the baseline survey happened many years in the past, it is also likely that economic development has occurred in a country and reduced the transmission intensity in many locations, which is another explanation why resurgence to baseline levels is not observed at the country level.

## Conclusion

Overall, our results suggest that treatment of WRA can achieve a small but significant decrease in morbidity and that this decrease can be obtained at very low cost: distribution cost will be near to zero and the cost of the tablets is a few cents per dose. Annual treatment of adolescent girls during HPV vaccine programmes has a similar impact on morbidity as deworming of pSAC/SAC in this age group. Ongoing SB treatment in combination with treatment of WRA may reduce morbidity in WRA, particularly in highly endemic areas and areas where infection levels are concentrated in adults. We therefore recommend that the impact of treating WRA on morbidity in this demographic should be monitored. Regardless of the population-level effectiveness of treating WRA, deworming is still expected to be beneficial to pregnant women and their children: STH infection of mothers during pregnancy has been shown to correlate with an increased risk of anaemia, which increases the risk of pregnancy complications [3, 26], and deworming in antenatal clinics has been shown to reverse this trend [27]. In addition, antenatal treatment increases birthweight and reduces mortality [28, 29] and the risk of STH infection in very young children (0–3 years) [25]. At this young age, STH infection can have a severe impact on child development.

## Supplementary Information

**Additional file 1: Table S1.** Model parameters and data sources.

**Additional file 2: Figure S1.** Prevalence of any infection in girls/women as they age.

## Data Availability

Data sharing not applicable to this article as no datasets were generated or analysed during the current study. The code that has been used for the simulation study has been published and can be accessed at https://github.com/caro-veg/STH_WRA_Morbidity.

## Abbreviations

MDA: Mass drug administration
M&HI infections: Moderate- and high-intensity infections
pSAC: Pre-school-age children
SAC: School-age children
SB: School-based
STH: Soil-transmitted helminths
WHO: World Health Organization
WRA: Women of reproductive age
