# Supplementary material for: Deworming women of reproductive age during adolescence and pregnancy: what is the impact on morbidity from soil-transmitted helminths infection?
Source: Parasit Vectors. 2021 Apr 23;14:220. doi: 10.1186/s13071-021-04620-w (PMC8063329; doi:10.1186/s13071-021-04620-w)
Supplement: Supplementary file 1 — Additional file 1: Table S1. Model parameters and data sources. [file 13071_2021_4620_MOESM1_ESM.docx]

Table S1: Parameter values used in the stochastic individual-based simulations for the EMC (Erasmus MC) and ICL (Imperial College London) models of hookworm transmission.

| Parameter | EMC | ICL |
| --- | --- | --- |
| Human demography | Demographic data taken from 2003 Kenya Demographic and Health Surveys (1) | Demographic data taken from 2003 Kenya Demographic and Health Surveys (1) |
| Aggregation of parasites in host k_w_ | 0.35 (2) | 0.35 (2) |
| Variation in exposure and contribution to the environmental reservoir by age | Relative exposure and contribution to the reservoir both increase linearly from 0 to 1 between ages 0–10 and is stable thereafter with no difference between males and females (3, 4) | Relative exposure and contribution to the reservoir are assumed to be equal in all age groups based on fitting the model to prevalence and infection intensity data from the TUMIKIA and DeWorm3 studies (5) |
| Average worm lifespan | 3 years (6-8) | 2 years (9) |
| Variation in worm lifespan | Weibull distribution with shape 2, i.e. the mortality rate is zero at age zero and then increases linearly with worm age (3) | Exponential distribution, i.e. the mortality rate is constant and independent of worm age |
| Pre-patent period | 7 weeks (4, 6, 7, 10) | No pre-patent period used |
| Age-dependent reproductive capacity of the worm in the human host | Constant over age (assumption) | Constant over age (assumption) |
| Female worm fecundity | Density-dependent on total number of female worms in host, assuming hyperbolic saturation (3)  On average 8.3 eggs per female worm per 41.7 mg sample of faeces (200 epg per female worm, as previously reported based on association between number of expulsed adult female worms and egg counts based on Kato-Katz (11)). The average maximum total host output is assumed to be 62.5 eggs per 41.7 mg faeces (1500 epg, as previously assumed (3)) | Density-dependent on total number of female worms in host, assuming exponential saturation. Exponential model of saturation with parameter γ = 0.02 (12)  On average 3 eggs per female worm per 41.7 mg sample of faeces (72 epg per female worm, as previously reported based on association between number of expulsed adult female worms and egg counts based on Kato-Katz (11)) |
| Survival of infective material in the central reservoir | Exponential survival (assumption)  Average lifespan of two weeks, implemented as a monthly survival probability of exp(-26/12)=11.5% (95%-CI: 0.05–7.38 weeks under assumption of exponential survival), based on the notion that average survival time is in the order of weeks (4, 10, 13) | Exponential survival (assumption)  Average lifespan of 30 days (9) |
| Proportion of adult worms killed by single dose of albendazole (400 mg) | 0.95 (14) | 0.95 (14) |
| Variability in measured host load of infective material (eggs per examined sample of faeces) k_egg_ | Kato-Katz: negative binomial distribution with aggregation parameter k=0.35, estimated separately from repeated individual-level egg count data from Uganda (15) | Kato-Katz: negative binomial distribution with aggregation parameter k=0.35, estimated from unpublished triple egg count data from Tamil Nadu, India |
| Cut-offs for no, light, moderate, and heavy infection | 1, 2000, and 4000 epg (16) | 1, 2000, and 4000 epg (16) |

1. Central Bureau of Statistics CBSK, Ministry of Health MOHK, Macro ORC. Kenya Demographic and Health Survey 2003. Calverton, Maryland, USA: CBS, MOH, and ORC Macro; 2004.

2. Bradley M, Chandiwana SK, Bundy DA, Medley GF. The epidemiology and population biology of Necator americanus infection in a rural community in Zimbabwe. Transactions of the Royal Society of Tropical Medicine and Hygiene. 1992;86(1):73-6.

3. Coffeng LE, Bakker R, Montresor A, de Vlas SJ. Feasibility of controlling hookworm infection through preventive chemotherapy: a simulation study using the individual-based WORMSIM modelling framework. Parasites & vectors. 2015;8(1):541.

4. Brooker S, Bethony J, Hotez PJ. Human hookworm infection in the 21st century. Advances in parasitology. 2004;58:197-288.

5. Truscott JE, Ower AK, Werkman M, Halliday K, Oswald WE, Gichuki PM, et al. Heterogeneity in transmission parameters of hookworm infection within the baseline data from the TUMIKIA study in Kenya. Parasites & vectors. 2019;12(1):442.

6. Bethony J, Brooker S, Albonico M, Geiger SM, Loukas A, Diemert D, et al. Soil-transmitted helminth infections: ascariasis, trichuriasis, and hookworm. Lancet (London, England). 2006;367(9521):1521-32.

7. Anderson R, Truscott J, Hollingsworth TD. The coverage and frequency of mass drug administration required to eliminate persistent transmission of soil-transmitted helminths. Philosophical transactions of the Royal Society of London Series B, Biological sciences. 2014;369(1645):20130435.

8. Truscott JE, Hollingsworth TD, Brooker SJ, Anderson RM. Can chemotherapy alone eliminate the transmission of soil transmitted helminths? Parasites & vectors. 2014;7:266.

9. Anderson RM, May RM. Helminth infections of humans: mathematical models, population dynamics, and control. Advances in parasitology. 1985;24:1-101.

10. Hotez PJ, Brooker S, Bethony JM, Bottazzi ME, Loukas A, Xiao S. Hookworm infection. The New England journal of medicine. 2004;351(8):799-807.

11. Anderson RM, Schad GA. Hookworm burdens and faecal egg counts: an analysis of the biological basis of variation. Transactions of the Royal Society of Tropical Medicine and Hygiene. 1985;79(6):812-25.

12. Coffeng LE, Truscott JE, Farrell SH, Turner HC, Sarkar R, Kang G, et al. Comparison and validation of two mathematical models for the impact of mass drug administration on Ascaris lumbricoides and hookworm infection. Epidemics. 2017;18:38-47.

13. AUGUSTINE DL. INVESTIGATIONS ON THE CONTROL OF HOOKWORM DISEASE. VIII.: EXPERIMENTS ON THE MIGRATION OF HOOKWORM LARVAE IN SOILS*. American journal of epidemiology. 1922;2(2):162-71.

14. Levecke B, Montresor A, Albonico M, Ame SM, Behnke JM, Bethony JM, et al. Assessment of anthelmintic efficacy of mebendazole in school children in six countries where soil-transmitted helminths are endemic. PLoS neglected tropical diseases. 2014;8(10):e3204.

15. Pullan RL, Kabatereine NB, Quinnell RJ, Brooker S. Spatial and genetic epidemiology of hookworm in a rural community in Uganda. PLoS neglected tropical diseases. 2010;4(6):e713.

16. World Health Organization. Soil-transmitted helminthiases: eliminating soil-transmitted helminthiases as a public health problem in children: progress report 2001-2010 and strategic plan 2011-2020. Geneva: World Health Organization; 2012.
