# Supplementary material for: Deworming women of reproductive age during adolescence and pregnancy: what is the impact on morbidity from soil-transmitted helminths infection?
Source: Parasit Vectors. 2021 Apr 23;14:220. doi: 10.1186/s13071-021-04620-w (PMC8063329; doi:10.1186/s13071-021-04620-w)
Supplement: Supplementary file 2 — Additional file 2: Figure S1. Prevalence of any infection in girls/women as they age. [file 13071_2021_4620_MOESM2_ESM.docx]

**Additional file Figures**


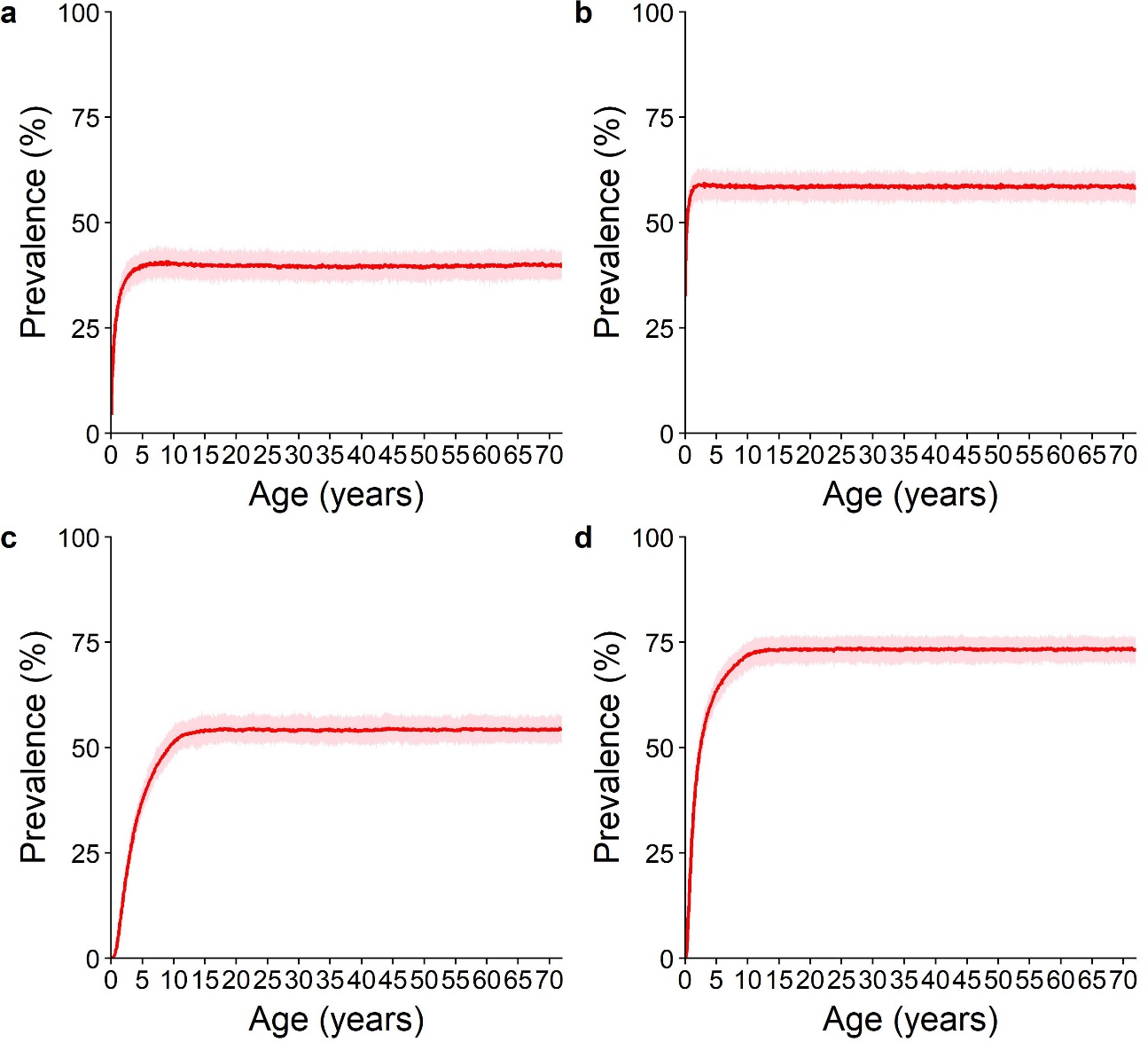


**Figure S1**: Prevalence of any infection in girls/women as they age in the absence of any treatment for STH. Red line: mean; pale red area: 95% credible interval. a, c: Moderate baseline prevalence (20-50%). b, d: High baseline prevalence (>50%). a, b: Imperial College London (ICL) results. c, d: Erasmus MC (EMC) results.
